# The effectiveness and cost-effectiveness of telephone triage of patients requesting same day consultations in general practice: study protocol for a cluster randomised controlled trial comparing nurse-led and GP-led management systems (ESTEEM)

**DOI:** 10.1186/1745-6215-14-4

**Published:** 2013-01-04

**Authors:** John L Campbell, Nicky Britten, Colin Green, Tim A Holt, Valerie Lattimer, Suzanne H Richards, David A Richards, Chris Salisbury, Rod S Taylor, Emily Fletcher

**Affiliations:** 1 Primary Care Research Group, University of Exeter Medical School, Exeter, EX1 2LU, UK; 2 Institute of Health Service Research, University of Exeter Medical School, Exeter, EX1 2LU, UK; 3 Department of Primary Care, Health Sciences, University of Oxford, Oxford, OX1 2ET, UK; 4 School of Nursing Sciences, Faculty of Medicine and Health Sciences, University of East Anglia, Norwich, NR4 7TJ, UK; 5 Mood Disorders Centre, College of Life and Environmental Sciences, University of Exeter, Exeter, EX4 4QG, UK; 6 Academic Unit of Primary Health Care, School of Social and Community Medicine, University of Bristol, Bristol, BS8 2PS, UK

**Keywords:** Primary care, Telephone triage, Decision support, General practitioner, Nurse, Workload, Satisfaction, Cost-effectiveness, Cluster randomised controlled trial

## Abstract

**Background:**

Recent years have seen an increase in primary care workload, especially following the introduction of a new General Medical Services contract in 2004. Telephone triage and telephone consultation with patients seeking health care represent initiatives aimed at improving access to care. Some evidence suggests that such approaches may be feasible but conclusions regarding GP workload, cost, and patients’ experience of care, safety, and health status are equivocal. The ESTEEM trial aims to assess the clinical- and cost-effectiveness of nurse-led computer-supported telephone triage and GP-led telephone triage, compared to usual care, for patients requesting same-day consultations in general practice.

**Methods/design:**

ESTEEM is a pragmatic, multi-centre cluster randomised clinical trial with patients randomised at practice level to usual care, computer decision-supported nurse triage, or GP-led triage. Following triage of 350–550 patients per practice we anticipate estimating and comparing total primary care workload (volume and time), the economic cost to the NHS, and patient experience of care, safety, and health status in the 4-week period following the index same-day consultation request across the three trial conditions.

We will recruit all patients seeking a non-emergency same-day appointment in primary care. Patients aged 12.0–15.9 years and temporary residents will be excluded from the study.

The primary outcome is the number of healthcare contacts taking place in the 4-week period following (and including) the index same-day consultation request. A range of secondary outcomes will be examined including patient flow, primary care NHS resource use, patients’ experience of care, safety, and health status.

The estimated sample size required is 3,751 patients (11,253 total) in each of the three trial conditions, to detect a mean difference of 0.36 consultations per patient in the four week follow-up period between either intervention group and usual care 90% power, 5% alpha, and an estimated intracluster correlation coefficient ICC of 0.05. The primary analysis will be based on the intention-to-treat principle and take the form of a random effects regression analysis taking account of the hierarchical nature of the study design. Statistical models will allow for adjustment for practice level minimisation variables and patient-level baseline covariates shown to differ at baseline.

**Trial registration:**

Current Controlled Trials ISCRTN20687662

## Background

Demands on UK primary care are escalating. The introduction of a new General Medical Services (nGMS) contract in 2004 [1] was followed by an estimated 25% increase in workload [2], requiring alternative ways of managing patient and government expectations and delivering safe, high quality care. Telephone triage has become widely adopted across the UK over this period. The introduction of the Walk In Centre (WIC) and the 24-hour nurse-led telephone advice service, National Health Service (NHS) Direct, the increased diversity of skill mix, and the use of remote consultations in primary care all represent organisational responses aimed at increasing the range of services available and improving access. When combined with telephone consultation, telephone triage provides rapid access to healthcare advice whilst freeing up opportunities for face-to-face consultation. Previous research [3] has demonstrated the utility of nurse-led telephone triage of patients requesting same-day appointments in UK general practice. An average practice (7,000 patients) might be expected to manage around 20 patients each day requesting a same-day appointment, representing around 35% of the General Practitioner (GP) workload [4].

Some research evidence exists regarding the feasibility, workload implications and cost of telephone triage, and patient experience of care, safety and health status following telephone triage. Most evidence derives from models involving nurse triage; less research has been carried out addressing the value of GP telephone triage. There have been no large scale multi-practice studies examining the potential value of nurse- or GP-led telephone triage of patients requesting same-day consultations.

### Feasibility

Previous studies suggest that around 50% of nurse triage calls may be handled by telephone advice alone [5-8]. However, such studies have been small or focused on out-of-hours care. Use of telephones (fixed or mobile) is now virtually universal in Britain [9], and recent years have seen a near quadrupling in the proportion of GP consultations conducted on the telephone, from 3% (1995) to 11% (2007) [10,11].

### Primary care workload

In the short term, telephone triage, whether by doctor or nurse, appears to reduce GP workload by around 40% [3,12], but may be associated with an increase in later return consultations of a roughly similar magnitude (30% [3], 50% [13]), in effect smoothing out the peaks and troughs of GP workload associated with same-day appointment requests but raising concerns regarding patient safety, convenience and cost-effectiveness.

### Cost

Equivocal results on costs have been seen across three studies. NHS Direct nurse triage has been found to be more expensive than practice nurse triage of patients making same-day consultation requests [14], but similar costs have been reported elsewhere for standard management and practice nurse-led triage of same-day consultation requests [3]. Nurse-led triage out of hours may reduce long-term NHS costs, but may not be cost-effective at all times of the day [15,16].

### Patient experience of care

Equivocal results on acceptability and satisfaction have been derived from small studies. One study [13] reported no difference in satisfaction between telephone and face-to-face consultations. Jiwa [12] reported that 80% of patients were satisfied with GP telephone management of same-day consultation requests, and Brown and Armstrong [17] have suggested that patients who use GP telephone consultations may do so as an alternative to face-to-face consultations in primary care.

### Patient safety

Telephone consultation appears safe and effective [18,19] although one Swedish study [20] noted that nurses often used self-care advice and over-rode software-determined recommendations on management. A recent Dutch study highlighted concerns regarding information gathering in telephone triage when delivered without interactive computerised decision support, relying only on clinical protocols [21]. Studies [8,20,22,23] have highlighted the importance of training in the use of decision support software in addressing patient safety issues. Nurse telephone triage using national guidelines (but not with interactive computer-based decision support) was judged to be efficient, although some concerns have been raised in respect of patient safety [24]. One study [3], adopting a triage system involving computerised management protocols developed by the practice, identified a 30% increase in the number of return consultations within the nurse telephone triage group, and although actual numbers were small, a substantial increase (200%) in Accident and Emergency (A&E) attendance. Although computerised, such a system did not provide interactive decision support (Richards D; personal communication) such as is now available within a number of NHS primary care computer systems and which we propose to examine in this study [25]. The other trial by Richards *et al*. [14] used computerised decision support algorithms for NHS Direct triage nurses, but not for nurses acting in primary care. A systematic review [26] of nine studies of telephone consultation and triage identified the possibility that telephone management may lead to a delay in providing definitive care.

### Patient health status

Several randomised studies (but none involving telephone triage) have compared the health status of primary care patients following consultations with a doctor or a nurse by patients with minor problems or after a same-day consultation request. One study [27] identified no difference in Short Form (SF)-36 scores between the intervention groups when followed up after two weeks. Similar findings have been reported in respect of resolution of symptoms and concerns after two weeks [28], or in the proportion of patients reporting themselves as cured or improved two weeks after a consultation with either a doctor or a nurse [29].

### Outcome measures

The identification and choice of the most relevant outcomes has been a contentious issue in previous evaluations of triage systems in primary care [26]. Most studies to date have assessed primary care and hospital service use and workload. However, these outcomes may not in themselves capture the aim of triage services, and more broadly, the aim of primary care. In the context of patients presenting requests to be seen on the same day, we propose that the purpose of a primary care consultation management system is to provide an administrative framework for practices (i) to facilitate the safe, timely, and definitive (first pass) management of such patients and (ii) to facilitate the timely and efficient management of primary care consulting time resource. Our proposed outcome measures (below) reflect that understanding. Additionally, it may well be that a large-scale study to inform the optimal management of same-day requests will ultimately benefit other providers (for example, out-of-hours GP service of NHS 24 Direct) that may steer patients towards general practice for semi-urgent problems.

UK-based trials of primary care telephone consultation and triage [3,5,12,13] have been conducted in relatively small populations and/or in limited numbers of practice settings, and without the use of computerised interactive decision support. Despite uncertainty about the benefits and costs, many practices operate GP- or nurse-led triage systems of different types as a way of providing fast access to care for patients and in order to manage practice workload. The NHS Institute for Innovation and Improvement has recently promoted a new model of GP-led telephone triage [30], the Stour access system, but without any robust evidence about benefits. We therefore propose to address this important agenda for both UK and international primary care in a large-scale experimental study of two forms of triage currently being promoted by the NHS for use in UK Primary Care. Our findings may be generalisable to other health settings, especially those with strong primary care-based health care systems.

### Aims

The overarching aim of this trial is to assess the clinical- and cost-effectiveness of nurse-led, computer-supported telephone triage and GP-led telephone triage, compared to usual care for patients requesting same-day consultations in general practice. Specific objectives of this trial are to compare the effects on primary care workload and cost, and patient experience of care, patient safety and health status, of: (1) nurse-led, computer-supported telephone triage versus usual care for patients requesting same-day consultations in general practice; (2) GP-led telephone triage versus usual care for patients requesting same-day consultations in general practice, and (3) nurse-led computer-supported telephone triage versus GP-led telephone triage.

### Pilot study

A preliminary pilot randomised controlled trial (RCT) was conducted in six practices to: (1) confirm the implementation of the GP-led and nurse-led triage systems as feasible; (2) confirm the proposed recruitment of practices and refine data collection systems, and (3) confirm the assumed level of clustering of outcomes. Aspects of this 12-month pilot study were informative to the final main trial protocol and we note these within the protocol below.

## Methods/design

### Design

The study is a pragmatic, three-arm, cluster RCT. Practices will be randomised 1:1:1 to receive GP-led triage, nurse-led computer based triage, or usual practice.

### Interventions

#### GP-led telephone triage

We will use components of the Stour Access System to deliver GP-telephone triage [30] as designed in 2000 by a four-partner teaching practice in Christchurch, Dorset, UK. A core element of the intervention is the use of the GP to undertake triage (as opposed to other non-medical members of the clinical team) for patients registered with their practice. Whilst Stour’s general model is that all appointment requests are triaged, in this study we focus only on those patients seeking same day consultations.

Stated success factors of the Stour GP-led triage system [30] include patients who did not attend appointments (DNAs) reduced to almost zero (from estimated national average of 6.5% to 7.7% [31,32]) and one-third of patients being dealt with over the phone without needing to see a member of the practice team. However, these claims are not yet supported by any other published evidence.

The triage component of the Stour Access system operates as follows. Once the receptionist has established that the patient is requesting a same-day appointment, the patient is asked to leave a contact number with the receptionist and is advised that the GP will call them back within around 1 to 2 hours. This timescale (for both the GP-led and nurse-led arm) is flexible, so as to optimise prioritisation. The GP discusses the complaint with the patient and triages them to the most appropriate person, such as a nurse, or books a face-to-face appointment with the GP, or provides advice on the telephone. An appointment slot with the appropriate clinician is booked by the GP - the patient is not referred back to the receptionist for this. An integral part of the Stour system is that patients can book directly with the nurse or other non-GP members of the primary care team and can access a walk-in nurse clinic where the practice offers this.

The Stour Access system has an established framework to help general practices to put the system into practice. This involves GPs beginning by auditing their face-to-face consultations over a single day to ascertain the number that needed to see their GP, those that could have been seen by a nurse, and those that could have been dealt with using telephone advice. Where appropriate, GPs are offered information about attending courses in telephone consultation, although it is recognised that most GPs are experienced in this. In the present study, guidance will be provided to GPs regarding dealing with patients effectively over the telephone. Practices are encouraged to consider language barriers for those with hearing or speech problems, or for those from diverse ethnic backgrounds who may not have English as a first language. They are also guided to consider their telephone infrastructure and space/ personnel implications of implementing the new access system. Lastly they should devise a timed system for GPs to call back patients and ensure that mechanisms are in place to inform patients about the new system.

This framework will be used as a template for practices involved in the GP-led arm of the trial and will be tailored to meet the trial aims and objectives and the needs of practices themselves.

#### Nurse-led computer-supported telephone triage

The Plain Healthcare Odyssey PatientAssess will be used to support nurses to deliver telephone nurse triage for patients registered at their practice. A computerised clinical decision support (CCDS) system will be used to assist nurses at the practice (Nurse Practitioners and Practice Nurses) in assessing and making decisions about the clinical needs of patients who have called their practice requesting a same-day appointment. This is a complex intervention involving training - both clinical and technology based; decision-support - to assess and plan care, and process and organisational change in practices, particularly in terms of reception activity and appointment system management. The package will be evaluated as a whole, in line with Medical Research Council (MRC) recommendations for evaluating complex interventions to improve health.

Odyssey PatientAssess is a UK product, developed to support nurses and paramedics to assess the clinical needs of patients. It is already being used by several out-of-hours and NHS walk-in services, and is also the subject of the ongoing Department of Health-funded trial, Support and Assessment for Fall Emergency Referrers (SAFER)-1 trial focusing on the care of older people who have called the 999 emergency service following a fall, and the Health Technology Assessment (HTA)-funded SAFER3 trial focusing on the care of adults who have called the 999 services and are not in need of transfer to an Emergency Department (ED).

Odyssey PatientAssess has been evaluated in a number of other RCTs, and its clinical and cost-effectiveness in settings other than in-hours general practice is already established. For instance, it has been demonstrated in one study involving a co-investigator [33] to be safe and cost-saving in the long term compared to GP telephone triage in a trial of out-of-hours consultations. That study remains the largest trial of nurse telephone triage to date. Furthermore, currently in excess of 60% of PCTs commission out-of-hours services that use nurses to triage patients by telephone, supported by Odyssey PatientAssess (personal communication, Chris Coyne, Plain Healthcare). However, the findings and experience of out-of-hours care (providing care for approximately 10.8 million contacts per year in the UK) cannot necessarily be generalised to the very different system providing in-hours primary care (approximately 1 million contacts per working day).

Odyssey provides the user with a network of assessment prompts and guided responses relating to over 465 presenting complaints. It allows for multiple symptoms to be evaluated simultaneously, supported by evidence-based frameworks for referral and self-care, with all assessment data remaining visible at all times. It supports the clinician’s judgment and expertise through enhancing normal consultation processes. The clinical database comprises several hundred assessment and examination guidelines and protocols, each linked to triage, treatment and advice guidelines, differential diagnoses, patient information and education. These are maintained by an in-house clinical development team that reviews the entire clinical content at least annually to ensure that it reflects current best practice, including National Institute for Health and Clinical Excellence (NICE) guidance.

The assessment screens include drop-downs that provide regularly up-dated referenced information on differential diagnoses and rationales for lines of enquiry for each type of presentation, so reminding the user about the importance of different lines of enquiry. For the purposes of the ESTEEM trial, Odyssey PatientAssess will be embedded within GP computing systems (Egton Medical Information Systems (EMIS, UK) and SystmOne (The Phoenix Partnership, UK)), or installed to run in parallel alongside all other systems (Synergy, Microtest, Vision). Odyssey PatientAssess guides and stores documented records of the assessment, advice and/or referral of each patient producing a fully auditable record. Based on the data elicited during the telephone assessment, Odyssey PatientAssess suggests an appropriate care plan (for example, patient advice, same-day appointment, home visit, routine appointment, 999 emergency referral).

Nurses allocated to the intervention group will receive additional training in the use of Odyssey PatientAssess and in telephone consultation skills. Following this there will be a pre-trial period of one month during which they will be expected to practise using the decision support in their daily work and, towards the end of this period, their use of the system will be assessed to ensure that they have achieved proficiency. The software is designed to support the nurse’s clinical decision making. Given the pragmatic nature of the trial, there will be no requirement in the trial that the advice is actually followed by the nurse or the patient. Plain Healthcare provided training and guidance to individual nurses until they felt ready to begin the trial and no nurses failed to achieve proficiency during the pilot study.

#### Training practices in the two triage systems

The precise model for training practices in each trial arm was devised during the pilot study. The training will be delivered by Plain Healthcare (nurse-led triage) and Productive Primary Care Ltd (GP-led triage) for the purposes of the trial. The structure for the training within the main trial is as follows: (i) initial whole-practice meeting with researcher to outline the trial, staff group involvement and timeline of training; (ii) 1-week audit by practice reception team of patient telephone requests for appointments to assess demand for same-day and pre-bookable appointments, and the collection by the research team of GP and nurse appointment capacity information; (iii) training for both GP- and nurse-led triage in organising the triage system based on demand and capacity data collected (Productive Primary Care); (iv) GP triage skills training for GP-led triage practices (Productive Primary Care); (v) training in professional issues and telephone consultation skills for nurse-led triage practices (Plain Healthcare) and (vi) briefing sessions given by the researcher to different practice staff groups, for example, clinicians, reception team, administrators/practice manager, in research procedures for the trial. In addition, nurses at nurse-led triage practices receive one-to-one remote training from a learning and development advisor from Plain Healthcare to provide support while they practise using Odyssey PatientAssess in the weeks leading up to the trial.

#### Usual care

Practices will be asked to continue with their standard consultation management systems for handling same-day consultation requests. We intend to describe in detail the systems used in usual care practices. All practices will be asked to complete a short questionnaire to collect details on their current staffing arrangements and systems to manage same-day consultation requests, including the extent to which they use triage of any sort, and their current appointment system arrangements.

Following training for intervention practices, practice systems will be allowed to stabilise to the allocated condition during a four-week run-in period prior to a five-week intervention and patient recruitment period.

### Patient recruitment

**Figure 1:**
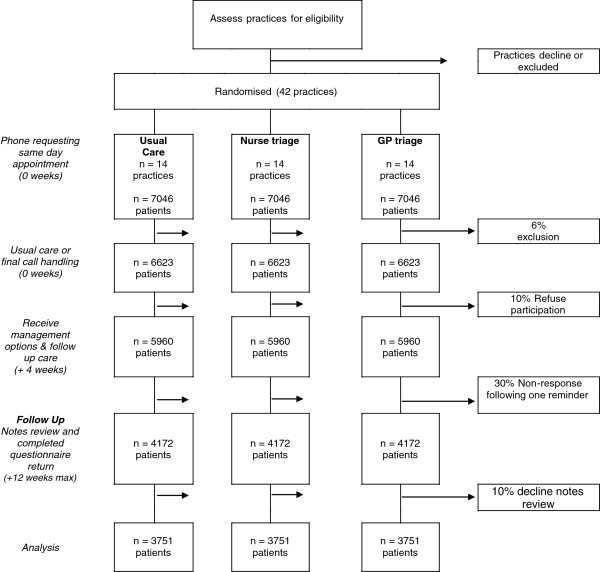
ESTEEM trial outline and patient flow.

During the intervention period all incoming calls will be received by the practice receptionist. Patients requesting a same-day consultation will initially be told the date of the first available routine appointment by the receptionist, and asked if this is soon enough. If patients continue to request a same day consultation, the receptionist will (i) flag the electronic record by means of a short keystroke entry code, noting the date and time of the same-day consultation request, which will automatically insert a read-code into the patient’s record, thus defining the eligible sample of patients; (ii) inform the patient of the current practice consultation arrangements; (iii) notify patients that a postal questionnaire regarding their experience of care may be sent to them after four weeks and that their help in completing this would be very much appreciated and (iv) manage the consultation request in accordance with the standard operating procedure for that practice. The consulting (triage/usual care) clinician will reiterate at the end of the clinical interaction that patients may be sent a questionnaire after four weeks and will ask patients whether they would be agreeable to a researcher reviewing their notes after twelve weeks. Patients would be given the opportunity to opt out of the notes review within the questionnaire sent four weeks later should they wish to do so at that stage (Figure 1).

### Inclusion and exclusion criteria

Practices already implementing a documented triage system for handling same-day appointment requests will be excluded. A documented triage system for the purpose of this study is defined as a system involving telephone triage (by a GP or a nurse) to manage more than 75% of all same-day requests. This definition recognises that a great number of GP practices will already undertake some form of telephone triage, and we will document the extent of any such triage in usual care practices as outlined previously. All consecutive patients making telephone requests for same-day consultations will potentially be included. Where an individual patient has multiple same-day consultation requests during the study period, to avoid confusion in following up the contact, only the first of these will be included in the study. We will provide guidance to practices on sampling throughout the working day. Larger practices (> 10,000 patients) may recruit up to 30 patients per day, compared with medium (20) and smaller (< 5,000 patients; 10 to 15) practices. All patients aged < 12 and ≥ 16 years requesting a same-day consultation will be included in respect of the primary outcome measure.

Parents or guardians of children aged < 12 years will be invited to provide consent on behalf of the child. We do not propose to include young people aged 12.0 to 15.9 years in this study, since this will involve receipt of a postal survey along with written consent to review notes to the young person’s address, a process we believe may inadvertently lead to a breach of confidentiality should third parties open or have access to the young person’s mail. Adults > 16.0 years will be included, unless the practice wishes to screen out patients for whom it would be inappropriate to send a questionnaire (for example, patients with recent bereavement, vulnerable adults, et cetera). Reception staff will invite patients making same-day consultation requests to briefly outline the nature of their problem in order to facilitate timely care. Patients seeking urgent care will be excluded from the study if they are (i) too ill to participate [29] (severe chest or abdominal pain or severe difficulty breathing; vomiting blood; altered consciousness; seizures; pregnancy related problems; or severe psychiatric symptoms); (ii) unable to speak English or (iii) a temporary resident.

### Practice recruitment

A total of 42 practices will be recruited from across four geographical areas (Bristol/Avon, Devon/Cornwall, Norfolk and Warwickshire/West Midlands South). To maximise recruitment we will seek to run the trial through the South West PCRN (Devon Primary Care Trust (PCT) is the host organisation and includes areas to Gloucester/Bristol and Central England PCRN (West Midlands South). Two of the four centres link directly to the South West PCRN, each centre having close links with the coordinating hub hosted in the University of Exeter Medical School. We will employ a two-stage approach to the recruitment of practices: (1) a written letter inviting participation to all practices in the four geographical areas, co-signed by the trial Principal Investigator, the local co-applicant, and the local National Institute for Health Research (NIHR) PCRN clinical lead and (2) recruitment workshops for those practices indicating an interest in participation, where the trial design and methods will be explained and provide an opportunity for practices to clarify their questions.

We are not complacent regarding the challenges of practice recruitment, recognising high-level evidence on the challenges of recruitment to large scale clinical trials of complex interventions [34]. Building on the experience of a previous limited survey, during the pilot phase we undertook a further survey of practices seeking expressions of interest in participation. We will provide a carefully presented recruitment pack to practices expressing preliminary interest; we anticipate doing this during personal briefing meetings (for which the practice would be remunerated) rather than as mailed material. We anticipate writing to 600 practices with a project outline, meeting with 125 (20%) practices that have expressed interest in the study in screening meetings (either jointly or individually), and securing participation from around one-third of these (42 practices). The PCRN in the South West, Central (West Midlands South) and East of England covers approximately 1,500 practices. We will prioritise practices in workable proximity to recruitment centres (taking account of relevant sampling issues). Regarding generalisability, it is recognised that only practices willing to participate can be included in the trial. Although some practices may not wish to participate in a complex RCT where they have no choice about which arm they would be allocated to, it may be that many would be prepared to instigate a triage system that had been shown to work in a high quality study.

### Randomisation

Individual patient-level randomisation is not practical, does not reflect the practice-wide reality of triage system implementation and is vulnerable to contamination. Instead we will use a cluster RCT design [35]. Patients in the usual care group may be influenced by system-level changes such as the changed availability of different types of appointments and changes to the process of care. Randomisation will be carried out by an independent statistician at the Peninsula Clinical Trials Unit and codes held remotely. The sequence of randomisation will be computer-generated and stratified for geographical location, practice deprivation and practice list size. Stratification by locality will ensure that we have a similar number of standard care/GP/nurse triage practices in each geographical area. Simple randomisation will be used for the first twelve practices and then minimisation applied to ensure balance of stratification variables across the three arms.

### Outcome measures

The terms, telephone consultation, and triage, have often been used interchangeably in research reports [26]. Whilst triage is a concept drawing on ideas of prioritising and rationalising workload, we have already referred to the uncertainty in the academic literature of the relationship between triage and the assessment/management of patients. Triage concerns prioritising the use of resources and determining the necessary speed of response. Telephone consultations may also have other benefits in terms of easier access to care for patients. Our study will therefore incorporate these important outcomes: (1) total primary care workload (primary outcome measure, including primary care, GP out-of-hours, A&E and walk-in centre attendance); (2) NHS resource use/cost and non-attendance rates in primary care and (3) patient experience of care, patient safety, and patient health status.

### Primary outcome

The primary outcome measure is the number of healthcare contacts taking place in the four-week period following the index same-day consultation request. The number of contacts will include the initial clinical assessment contact, and will thus include the triage contact in the two intervention conditions. The total number of contacts per 1,000 patients requesting same-day consultations in each of the trial conditions will be derived from numbers of GP, practice nurse, GP out-of-hours, A&E, and (although they may not impact significantly on GP workload [36]) NHS WIC attendances. Data collection, capturing information on timing (am/pm), and type (face-to-face, telephone, home visit, GP out-of-hours, A&E and WIC attendance) of primary care contacts in all trial practices will take place as part of a note review occurring at least twelve weeks after the index consultation request with data extracted from the primary care medical record. We will count the day of the index request as day 1 (same day), and include the following 28-day period (day 1 to day 28 inclusive) starting from the day following the consultation request.

### Secondary outcomes

1.Descriptive study of patient flow: we will describe the management and interim and final disposition of patients in the working day in which they request a same-day consultation, up to the point of final contact within that working day, for each of the trial conditions.

2.Primary care NHS resource use: we will capture NHS primary care resource use by collecting data on the number of contacts resulting for patients in the three trial conditions, as for primary outcome, over a four-week follow-up. We will collect actual consultation length for initial triage and usual care (face-to-face or telephone) consultations (described below). We will estimate the length of subsequent consultations, for same-day and four-week follow-up periods based on the recorded start and end times of a sample of consultations captured over two randomly selected days in each practice during the patient recruitment period. For costs, see health economic evaluation below. Non-attendance rates for allocated appointments in the month following the same-day request will be described and compared between trial conditions using data extracted at practice record review.

3.Patient experience of care, safety, and health status: these patient-reported outcomes will be collected by postal questionnaire four weeks following the request for a same-day consultation. This time frame has been selected to capture a period for optimal recall, which may encompass events and experiences that could feasibly be linked to the initial reason for contacting the GP practice for a same-day consultation.

For patient experience of care, whilst this study focuses on patients’ experiences of an episode of care delivered following a same-day consultation request, and possibly involving multiple healthcare contacts during the course of the index day, currently available survey instruments suitable for use in a UK setting have focused on the evaluation of individual consultations [37,38] or aggregate (practice-based) care. No validated instruments for assessing the patient’s experience of an overall episode of care following such a request, and possibly involving multiple interactions, have been identified. We propose to use a modification of the new national GP patient survey instrument for the purposes of monitoring patient experiences of care in this study. This study team is in a unique position [39,40] to have early access to this instrument for research purposes.

We will survey patients who have requested same-day consultations in each of the three trial conditions using a modified version of the national survey instrument. Whilst the national survey explores patients’ experiences of care in the past 6 months, we will modify relevant questions to focus on the participants’ recent experiences of care following their same-day consultation request.

The responsiveness of the consultation management system will be assessed using patient reports of care received (for example, how quickly care was provided and overall satisfaction) and their evaluations (using a 5-point Likert scale) [41,42] of the timeliness and convenience of the response [43] to the original same-day consultation request incorporated into the follow-up questionnaire.

For patient safety, deaths within 7 days of the same-day consultation request (from practice records), and attendance at A&E within four weeks and number and length of stay of emergency (unplanned, that is, admission with no evidence of prior planning of that admission within a one-day period at notes review) hospital admissions within 7 days of the index consultation (from primary care records examined twelve weeks after the same-day consultation request).

Patient health status will be assessed by the widely used EuroQol Group 5-Dimension Self-Report Questionnaire (EQ-5D) measure [44] incorporated into the follow-up questionnaire, along with a question on problem resolution [27,28] (5-point Likert scale).

For descriptive data, we will collect patient-level (for example, on age and gender) and practice-level (for example, on practice size, staffing and deprivation index) data. Two approaches to defining patient casemix will be adopted: (i) we will record morbidity on the basis of the principal systems involved (after [28,29]), allowing for clinicians to record up to three systems in respect of each consultation; (ii) an 8-point score will be used to define the complexity of the case (after Howie [45]) with each consultation being defined as having substantial (2 points), attributable (1 point) or no content (0 points) in respect of each of four domains, namely, physical, social, psychological or other (for example, administrative) components to the consultation.

Timely care will be assessed as (i) proportion of participants receiving initial advice in any form from the practice (that is, not including out-of-hours care, WIC or A&E attendance) the same day (that is, within routine surgery opening hours) following a same-day appointment request and (ii) delay (days) to final episode of care in the week following the index consultation request. Definitive first pass management will be assessed as the number of consultations (including index triage consultation) in the week following the index consultation request; patient reports of problem resolution and patient-reported experience of care.

Efficient management of consulting time will be assessed by examination of the extent to which additional consultations introduced as a result of GP or nurse triage are offset by altered patterns of subsequent consultations by the patient in (i) the same day as the request; (ii) the week following the request and (iii) the 28 days (four weeks) following the request; we will count the day of the index request as day 1 (same day), and in respect of (ii) and (iii) include the following 7-day period (day 1 to day 7 inclusive) or 28-day period (day 1 to day 28 inclusive) within these defined time periods.

### Blinding

Given the nature of the interventions it is not possible to blind patients or practitioners to treatment allocation. Although the cluster design of the trial might theoretically allow the researchers undertaking case note review to be blinded, our pilot study showed this was not possible in practice. The data analysis will be carried out by a statistician who is blind to treatment allocation.

### Sample size

Our estimation of sample size is based on the primary outcome of the number of healthcare contacts taking place in the four-week period following the index same-day consultation request. The estimation draws on a previous UK study comparing nurse-led telephone triage to standard practice for handling same-day consultation requests [3]. That trial reported the number of NHS consultations based on general practice (GP and nurse), A&E and out-of-hours contacts. We believe this to be a good proxy for our primary outcome. Over the four-week follow-up of the trial the authors reported a mean number of NHS consultations of 1.02 (pooled SD 0.78) (Table four in [3]) in usual care compared to a mean of 1.38 (pooled SD 1.79) in the nurse telephone-triage arm. Estimated trial sample size requirements for this trial are summarised in the table below. Four sample size scenarios are shown based on 80% or 90% statistical power and a intracluster correlation coefficient (ICC) of 0.01 or 0.05 (chosen as they represent the upper end of the average ICC reported from a survey across a range of outcome measures collected in trials from primary care settings [46]) (see Table 1).

**Table 1:** Sample size calculation

Number per group*	**ICC**	**Design factor**	**Number of patients per group**	**Final number of patients per group+**	**Number of practices per group**
305*	0.01	3.26	994	1,867	4
305*	0.05	12.3	3,751	7,046	14
228^	0.01	3.26	743	1,396	3
228^	0.05	12.3	2,804	5,267	10

*At 90% power and 5% alpha; ^80% power and 5% alpha (both using the above means and SDs as per text); +after 6% [3,27,28] of patients being excluded, 10% [14,28,29] initially declining participation, 30% [27-29] non-response to the request to return written consent to note review; of responders, 10% decline note review.

Selecting the most conservative sample size scenario (that is, 90% power, ICC 0.05) we estimate we require 3,751 patients per group to detect a difference of the magnitude reflected above (1.02 (0.78) versus 1.38 (1.79) primary care contacts in the follow-up period) in the primary outcome between the nurse-led triage and usual care arms. In order to inform the power study, and prior to undertaking the one-year pilot study (including the requirement for patient-written, rather than verbal consent), which would yield supporting data, we estimated that of all patients requesting a same-day consultation, 6% [3,27,28] will be deemed ineligible, 10% [14,28,29] will initially decline participation in the trial and/or follow-up of their notes, and 30% [27-29] will not respond to the request to return written consent to note review. Of those who do respond in the questionnaire survey, we estimate 10% (conservative) will decline note review. Thus a total of 7,046 patients seeking same-ay consultations across 14 practices over a five-week period will be required in each of the three trial arms (see Figure 1). In the absence of a published effect size for the primary outcome for the GP-led triage arm, we estimated the sample size requirement on the claimed impact of a 30% reduction in GP workload with the Stour Triage system, and assumed a reduction of 30% in the primary outcome. At 90% power and an ICC of 0.05, a total of 1,983 patients in the GP-triage and usual management arms would be required. However, given the uncertainty of the estimate of a 30% reduction, we propose to recruit the same number of patients requesting same-day consultations as estimated for the nurse-triage arm, that is, 7,046 patients (and 42 practices).

The pilot study provided confirmation of our assumed ICC of 0.05 (that is, 0.03, 95% CI {AU query: CI ok as added?}0.00 to 0.08). Furthermore, the pilot study, led to a change in the method of patient consent to note review from written consent (obtained from completed patient questionnaires) to include verbal consent obtained from the treating clinician. The proportion of patients agreeing to case note review is anticipated to increase from an estimated 63% (original power study) to an estimated 78% under revised, and ethically approved consenting arrangements.

### Feasibility

An average practice (7,000 patients) accommodates around 714 consultations per week [10] - approximately 142 consultations per day. Some [4] have estimated that up to one-third of these are patients seeking same-day consultations; however, case definition is of importance, and we believe that an estimate of around 20 patients per day, 100 per week (14% of workload) clearly seeking same-day consultations is conservative, and reasonable; this was confirmed as realistic following our pilot and feasibility study. To achieve our sample size, we will need to recruit patients in 42 practices, each for a five-week period of patient recruitment. It is acknowledged, however, that practices vary in terms of the number of same-day consultation requests they receive (for instance due to practice size, nature of local population et cetera). To reduce the likelihood of this leading to significantly uneven patient recruitment across the different practices, in practices where the number of same-day consultation requests is significantly above the average, we will randomly sample sufficient numbers of patients from each practice to ensure proportionality across the practices.

For the patient-related outcomes, self-reported health status and perception of access to care will be collected by postal survey distributed from the patient’s registered GP using practice-headed notepaper, and with an estimated response rate after two reminders of 70% [47-50]. The survey will be distributed four weeks following the index episode of care, to patients who have been identified for the trial. The survey response pack will incorporate written consent to review of the patient’s medical record with a view to capturing primary outcome measure data twelve weeks after the index episode of care. At 80% power and 5% alpha, our sample size of 7,046 per group will allow us to detect an effect size of 0.18 of a standard deviation at an ICC of 0.01 and an effect size of 0.34 of a standard deviation at an ICC of 0.05. Thus, we believe that the proposed sample size will allow us to be able to detect a small to moderate effect size in our patient-reported secondary outcomes.

### Economic evaluation

Primary economic analysis will take a cost consequences analysis (CCA) approach, that is, we will estimate the additional cost associated with the introduction and use of (i) nurse-led computer-supported telephone triage, or (ii) GP-led telephone triage compared to usual care/practice in the management of patients requesting same-day consultations in primary care, and present the costs alongside the benefits (if any) identified in the trial. The CCA approach is regarded as a form of full economic evaluation [51] even though findings are not specifically summarised into an incremental cost-effectiveness ratio. Given the expected consequences of the interventions being compared, for example, any change in the type or nature of primary care index or follow-up contacts or change in NHS resource use at a patient level, we see CCA as the most policy-relevant approach for the economic analysis as it provides decision-makers with information in a clear way and allows them to consider the issue of value for money in a context-specific manner. The primary economic analysis will estimate the mean costs of care associated with the primary outcome and triage (where used) for each of the trial arms for a four-week follow-up period.

Economic analysis will be undertaken from the perspective of the NHS and Personal Social Services (PSS), as for the current NICE reference case [52]. The nurse- and GP-led triage systems being compared to usual care are expected to involve some organisational change and additional resource use. These intervention costs will be estimated using within-trial case report forms from each participating general/primary care practice, to identify the additional components of resource use, and to measure the additional resource use for each intervention, compared to usual care or practice. Within both) nurse-led computer-supported telephone triage and GP-led telephone triage there will be a cost associated with staff training and initial set-up. Training log sheets will be used to accurately capture the duration of all staff training associated with the trial by staff type. There will be capital costs associated with nurse-led computer-supported telephone triage (provided by Plain Healthcare), and these will be depreciated against the expected life of the capital investments. Finally, within nurse-led computer-supported telephone triage and GP-led telephone triage, there will be time spent by nurses and GPs respectively on triaging patients that will be captured by asking the clinician delivering triage to fill in a data collection instrument, the clinician form.

Trial outcomes will identify resource use for each participating patient (for example, nurse, GP, out-of-hours, A&E, WIC), and trial analysis will report differences between such resource use for each arm of the trial over a four-week follow-up period. Data on resource use (participant level) will be combined with credible unit cost data from published and/or NHS sources [53,54] to estimate the mean cost (as previously discussed) for each arm of the trial, and mean cost over the four weeks from (and including) the index consultation. Methods used for collection of resource use data, other than against primary trial outcomes, were explored and refined in the pilot and feasibility phase of the research, to ensure methods were feasible and acceptable to participating primary care practices.

All data sources and assumptions used in the economic analysis will be clearly presented. Parameter and structural uncertainty will be addressed using extensive sensitivity analyses (one-way, multi-way, scenario analyses, and probabilistic/bootstrapping approaches). Analysis will be undertaken to investigate health-related quality-of-life (four weeks after the index consultation) using the EQ-5D, however, in the absence of a baseline quality of life measure, such analysis will be exploratory. Further exploratory analysis will be undertaken to investigate the impact on cost of using micro-level costing for follow-up appointments. Such micro-level costs will be informed by obtaining duration data as described (Secondary outcomes). Results presented from the economic evaluation will allow decision makers (macro-, meso- and micro-level) to consider the relative merits of the interventions in a policy-relevant way.

### Data collection

#### Clinician data collection forms

Clinicians will complete a short data collection form (clinician form) at the time of the initial consultation following a patient’s same-day request (triage or usual care contact). This form captures details of the consultation including which health professional undertook the consultation, whether the patient did not attend, case-mix (see Secondary outcomes, Descriptive data), treatment and management options chosen (for example, ordering of tests, recommending subsequent appointment or referral), start and end time of the consultation and the patient’s response to the initial verbal consent to note review, which the clinician asks for at the end of the consultation.

#### Questionnaires

Patients will be sent a questionnaire four weeks after their index consultation. The questionnaire will include questions on satisfaction and health status (as discussed above). The covering letter will contain a section for patients who do not wish to participate or receive any further contact in relation to the study. These patients will be asked to send back a blank questionnaire in a prepaid envelope as an indication that they wish to have no further contact. These patients will not receive any further communication in relation to the study. The questionnaire will contain a section at the end, which will offer the opportunity for patients who are willing to complete a questionnaire, to opt out of having their case notes reviewed (for further details see the section, Informed consent). Those who do not respond will be sent two reminder letters with a second copy of the questionnaire two and four weeks after the first mail-out.

#### Case note review

Research staff will undertake a review of case notes of patients who have consented to have their notes reviewed (see below for further details of procedure for obtaining informed consent). This will take place a minimum of twelve weeks after their initial same day consultation. The case note review will be undertaken in the relevant GP surgery and patients’ notes will not leave the surgery. The purpose of the review will be to record information on the number and type of healthcare contacts including casualty contacts and hospital admissions, and length of consultations where this information is available. Laptops or memory sticks (encrypted as per NHS security requirements) will be used by researchers to record encrypted data in the research database. A standardised process will be adopted when searching notes.

Although it is not possible to blind research staff to the trial arm that a practice has been allocated due to the cluster design, a small validation study will be undertaken to explore the impact of lack of blinding on the outcome assessment process. During the feasibility study, a small sample of notes (30 from one practice) was independently examined by the four trial researchers, and the relevant data extracted. The inter-rater reliability of the data extraction process was assessed, and any divergence between assessors documented and resolved with a view to developing standardised procedures to guide the review process in the main trial.

### Statistical analysis

Analyses will also take into account the additional CONSORT guidelines for cluster-randomised trials and pragmatic trials [3,4]. The full combined statistics and health economics plan has been reviewed by the Trial Steering Committee and Data Monitoring Committee and is available from the authors. A summary of proposed analyses is provided below.

The primary analysis will be based on an intention-to-treat (ITT) principle, that is, analysis of all trial patients in practices according to random allocation. It is assumed that the vast majority of patients in the two triage arms will receive the triage intervention, with only a few being managed in other ways. For the usual care arm, there is no intervention beyond the management the patient would receive if the trial were not taking place and hence no patients can fail to receive the intervention. The primary analysis will take the form of a random effects regression analysis taking account of the hierarchical nature of the study design (that is, allocation by practice) and to allow for adjustment for practice-level minimisation variables (geographical location, deprivation level and size of practice) and patient-level baseline covariates shown to differ (based on descriptive data) at baseline. There is potential for clustering by clinician with the practices; the magnitude of this effect will be estimated and where necessary incorporated in the data analysis. However, this is a pragmatic trial where the intervention is being delivered at the practice level, rather than at the level of individual clinicians. The same primary analysis models will be fitted for the primary outcome, secondary outcomes and costs. A generalised linear model (GLM) will be fitted with the appropriate choice of family and link function, according to the type of data and its properties. The intracluster correlation coefficient (ICC) will be reported for all primary and secondary outcomes. The individual components of the primary outcome, the patient experience questions, and the five individual questions that comprise the EQ-5D will be reported for each group descriptively. A secondary (per protocol) analysis will be performed (for the primary outcome only) as a secondary analysis if there are sufficient patients (for example, > 1%) in the triage arms who do not receive triage. In such an analysis, these patients would be excluded, such that only patients who receive the triage intervention are included.

The influence of practice-level characteristics (for example, deprivation, location and list size) on the primary outcome will be investigated using interaction terms. Although the power to detect moderate subgroup interactions will be low, we are primarily interested in investigating the possibility of large interactions, which are qualitative (the direction of treatment effects varies between subgroups) rather than quantitative (the magnitude of the treatment effect varies between subgroups, but not the direction). This subgroup analysis will be restricted to the primary outcome.

Sensitivity analyses will be conducted to investigate the potential impact of missing data on the primary outcome only. These will include making different assumptions such as best-case scenarios (for example, assuming a patient who does not consent to a case notes review has zero healthcare contacts in the four-week period following the initial telephone call requesting a same-day appointment), as well as multiple imputation models. Available demographic characteristics (depending on whether questionnaire is completed) of patients who consent to the case notes review will be compared to those who do not.

The primary economic analysis will estimate the mean cost of care across each of the trial arms, to include triage (where used) and the items in the primary outcome. Economic analyses will be based on a micro-level costing estimate for the triage intervention, and the use of published unit cost data for other elements of resource use. Estimates of the cost associated with triage interventions will be based on incremental costs when compared to usual care, with any capital costs and/or training costs depreciated/spread over an appropriate time period in the primary analyses (with other time horizons for these costs explored in sensitivity analyses).

The economic evaluation will present between group comparisons of costs using the regression-based statistical methods described above. Mean participant-level cost estimates for the triage interventions will be presented, with 95% CI. Although cost data often does not follow a normal distribution and parametric tests are therefore not appropriate, if a sample has a large number of observations as in this case (circa 18,000), incorporating central limit theorem implies parametric tests are appropriate and may be used [9].

Sensitivity analyses will be undertaken to explore the implications of uncertainty in data used, and assumptions made within the analyses. Exploratory analyses will be undertaken to estimate the costs associated with same-day primary care resource use, using trial data from the primary analyses combined with unit cost data, and also using data collected within the trial on a sample of same-day GP and nurse contacts (triage arms and usual care) to inform a more micro-level estimate of time spent in primary care on the same day as the triage index consultation.

Emphasis will be placed on estimation rather than hypothesis testing. Where hypothesis tests are carried out, these will be at the 5% level for primary and secondary outcomes, and the 1% level for interaction terms. While we adjust *P*-value cutoffs for the three-arm nature of this trial (that is, three independent between-group comparisons) we will not adjust for multiplicity of outcomes as such methods are too conservative when outcomes are positively correlated, as they would be in this trial. However, all analyses will be planned a priori and reported in full. All analyses will be conducted using Stata v.11. No interim analyses are planned.

### Process evaluation

A parallel process evaluation will be conducted aiming to assess the acceptability of each of the two triage interventions to patients and practice staff as follows: to (i) describe how each of the interventions was implemented in different practices; recent guidance [55] has noted that complex interventions such as those being evaluated here may work best if tailored to local circumstances; (ii) assess patient and practice staff expectations and experiences of the two interventions or standard care, and their views of the acceptability of the interventions and (iii) develop possible explanations for why each intervention did or did not work.

We will use qualitative methods employing telephone or face-to-face interviews with patients and practice staff [56,57]. The research will be undertaken in a random sample of ten participating practices (four in each of the triage arms, two in usual care). We will seek to involve practices of various list sizes and location (urban, rural).

#### Patients

In the main trial, a clinician involved in the management of patients requesting a same-day appointment will be asked to identify patients who might be suitable to be approached and asked to take part in a home or telephone interview study. Patients will be identified within 2 to 3 days of seeking treatment, and a letter sent to them from their practice (including a participant information sheet) asking if they would be willing to consider taking part. Patients who are interested will be asked to complete a reply slip including their contact details, and return it in a prepaid envelope direct to the research team. A total of 15 interviews per trial arm are planned (total of 45 patient interviews) and where possible, interviews will take place within two weeks of the index consultation. To achieve this number of interviews, it may be necessary to approach approximately 90 patients across the trial arms.

Researchers will select a sample of those agreeing to be interviewed to form a maximum diversity sample, on the basis of age, gender and ethnicity. Potential participants will be telephoned, and given the opportunity to discuss the study with a researcher prior to agreeing to a date and time for an interview. Any participants who expressed an interest, but who were not selected to take part, will receive a letter thanking them for their interest and informing them that they will not be interviewed. As the analysis develops, theoretical sampling may also be used to investigate emerging theories. Written consent to participation will be obtained at interview. Patients will be interviewed at home and asked about the index consultation: their own story of that consultation and its antecedents and consequences, the acceptability of the system they experienced, and their perceptions of convenience and speed of access. It is anticipated that the interviews will last between 30 and 60 minutes; they will be tape-recorded with patients’ permission.

#### Staff

Five members of staff in each of the practices contributing to the process evaluation will be invited to participate in an interview conducted in their own practices while the intervention is being implemented to minimise recall bias. They will be asked about details of the setting up and running of the intervention in their practice, the acceptability of the intervention, problems occurring and how they were or were not solved, their general perceptions of the intervention, and their hypothetical willingness (or not) to continue using the intervention once the study is over, with reasons. Interviews will be tape-recorded with participants’ permission.

#### Data handling/analysis

Interview recordings will be transcribed. The transcriptions and recordings will be analysed using a thematic analysis in the first instance, using some of the techniques of grounded theory such as constant comparison and theoretical sampling [57]. The purpose of the analysis will be to describe patient and staff perceptions and experiences of the two interventions, and to generate explanations for their success or failure as perceived by different participants. A specialist software programme such as NVIVO or Atlas-ti will be used to organise the qualitative analysis and ensure its systematic analysis. The researcher will begin by coding transcripts using a coding scheme drawn up in collaboration with other team members.

The data analysis will begin with a descriptive thematic analysis, firstly covering topics relevant to the research questions and secondly, other topics raised by interviewees. We aim to conduct a more conceptual analysis, using methods such as constant comparison, to begin to formulate explanations for the descriptive findings. The analysis of patient interviews will be iterative, moving between data collection and data analysis to test emerging theories. It may, for example, emerge that particular groups of patients have particular expectations about same-day consultations that shape their experiences of the interventions, and this may require deeper exploration. The iterative nature of the qualitative analysis will arise from the staged nature of the trial and the fact that interviews are to be conducted with patients and staff participating at different points of the trial. It may also be necessary to explore reasons for differences in how the two interventions are experienced. The analysis of patient interviews may require knowledge from the staff interviews about how the intervention was implemented in individual practices. Care will be taken to identify and follow-up deviant cases which do not fit into emerging theories. Preliminary findings will be sent to interviewees if desired, for confirmation and correction. The analysis of staff interviews will involve thematic analysis as above. It will also involve analysis of any areas of emerging agreement or disagreement about what did or did not work, any observable conflicts and any differences of opinion between groups of staff (for example, receptionists and nurses).

At least one of the co-applicants (NB) will contribute to the qualitative analysis and writing up of the qualitative data. The outputs of the qualitative analyses will include descriptions of how each intervention was implemented and experienced by participants; assessments of the acceptability of each intervention and tentative explanations for the reasons underlying apparent successes or failures.

### Ethical arrangements

Multi-centre research ethics approval (MREC) and local research governance approval for the study was obtained prior to the beginning of the pilot and feasibility work. The study personnel, management group and independent Trial Steering Committee will ensure that the study is conducted within appropriate NHS and professional ethical guidelines.

### Informed consent

Practice recruitment arrangements are described previously. All patients requesting same-day consultations over the five-week recruitment period in the study practices will be eligible for inclusion in the study (subject to the age limits specified above).

When patients call the practice seeking a same-day consultation the receptionist will advise them (or the guardian of children aged less than 12 years) of the current practice consultation arrangements. Once it has been ascertained that the patient does not require urgent or emergency care, the receptionist will explain briefly that a trial is taking place and advise the patient that the practice would appreciate them participating in a questionnaire survey, which will be sent to them for their consideration within around four weeks. The consulting (triage/usual care) clinician will reiterate that patients may be sent a questionnaire to ask their opinion of the triage system/existing consulting arrangements, and will ask patients at the end of the clinical interaction whether they would be agreeable to a researcher reviewing their notes in twelve weeks time. The patient’s verbal consent will be noted on a data collection form. Four weeks later the practice will send patients an information sheet describing the study, a questionnaire and a prepaid return envelope. Participants may opt out of the notes review on the last page of the questionnaire, should they wish to do so at that stage. A name with contact details will be provided should patients require any further information and those who do not wish to complete a questionnaire will be encouraged to return their blank questionnaire in the prepaid envelope, and they will receive no further contact in relation to the study. Those who do not respond to the questionnaire will be sent a second and third mail-out of the questionnaire and information sheet two and four weeks later.

### Process evaluation

Informed consent will be gained from all participants agreeing to be interviewed for the process evaluation. All patients and NHS staff being interviewed (including telephone interviewees) will be provided with a research information sheet. A researcher will briefly introduce the study and will allow participants the opportunity to ask questions. A consent form will be provided for participants to sign. The researcher will also sign the consent form. The staff member will be given a copy of their signed consent form to keep, and a further copy will be retained by the researcher.

Once informed consent has been obtained, the researcher will seek the participant’s permission to tape-record the interview, explaining the reasons for doing so. If a participant does not wish the interview to be recorded, the researcher will make written notes of the interview. Participants will be reassured that neither the tape nor the handwritten notes will contain any personal identifying information and that nobody will listen to the tape or read the notes of the interview, except for members of the research team involved in transcribing and/or analysing the data.

The observational aspect of the process evaluation, which will take place during the feasibility study, involves the researcher observing and taking brief field notes of the triage systems as they are implemented. In practice, this is likely to involve a researcher being present whilst the relevant member of staff undertakes telephone triage. NHS staff will be made aware that this is occurring, provided with information about the study, and their verbal consent obtained.

### Confidentiality

All personal information obtained about patients or staff for the purposes of recruitment or data collection (for example, names, addresses, contact details, personal information) will remain confidential and be held in accordance with the Data Protection Act. Each patient included in the trial will be assigned a research number and all data will be encrypted and stored without the subject’s name or address. Electronic data will be held on a secure database on a password-protected computer at the University of Exeter Medical School, and paper-based information held in a locked filing cabinet in the research team office. Access to data will be restricted to the research team. Names and participant details will not be passed to any third parties and no named individuals will be included in the write-up of the results. The only time personal information would be passed to a third party would be if we considered there was risk of serious harm to a research participant, and normally this would only occur after discussion with the person concerned.

Regarding case note review, the clinical record will only be viewed where patients have given consent for this to take place (that is, those who have not opted out of this aspect of the study). Only individuals with official employment contracts involved in the study or NHS staff attached to the study practices will be involved in accessing patient records. Case note reviews will only take place within the GP surgeries, in order to obviate the need to remove any patient records. Any electronic data extracted from medical records will be encrypted and will not include the names of individuals concerned. Where data are temporarily stored on laptops or memory sticks, or attached to emails prior to transfer to the research offices, these media will be encrypted as per NHS security requirements. Researchers will adhere to any confidentiality agreements stipulated by the practice concerned.

### Safety of participants and researchers

It is important to consider safety aspects both of patients and personnel involved in the study. There are not thought to be any significant risks to patients or staff in the proposed trial or process evaluation. Where there are minor risks we have developed systems to ensure these are minimised as detailed below.

#### Minimising risk of delays to care for emergency cases or inappropriate triaging

The patients under investigation are those requesting same-day consultations in their general practice. Consequently some patients are likely to perceive themselves to have urgent or emergency health care needs. Before triage occurs, all patients will be asked a standard question to ascertain whether they require urgent or emergency care. Where there is an emergency, patients will be dealt with according to the practice’s usual protocol and such patients will not be eligible for participation in the trial. All other patients requesting same-day requests will be dealt with according to a standardised procedure.

The telephone triage itself will involve some patients who might otherwise have been seen in person by a clinician being dealt with on the telephone. It is therefore essential that this does not lead to patient care being delayed when same-day care is needed. The telephone triage systems have been designed to provide safeguards against inappropriate triaging. Both have been tested in previous studies and have been found to be safe (see above), and are already used within some GP practices in the UK. Clinicians and receptionists involved in this trial will have received training in the system they are using and will be provided with ongoing support from a clinical lead in the practice, and from the system designers themselves. The system relies on decision support software (Odyssey nurse triage system), and includes a competency test for all those using the system. Since the Stour system draws on clinical skills routinely offered by GPs, no competency test is proposed for this system, although guidance in the practicalities of telephone triage will be provided for this aspect of clinical practice.

#### Minimising risk associated with unexpected software or triage problems

The trial uses two systems for triaging patients, one of which relies on decision support software. A number of steps will be taken to minimise the risks associated with any unexpected problems in these systems and will serve as an early warning system to prevent serious problems occurring. Each practice involved in the study will assign two named clinical leads who will be the first point of contact within the practice and who will be able to deal with any immediate problems and to take corrective action. A form will be established for use in practices to alert the clinical leads and the trial manager to any problems arising. The trial manager will establish appropriate mechanisms to ensure regular contact with the practices, particularly during the run-in and early stages of patient recruitment. Software, which is installed as part of the trial will be thoroughly tested before the trial begins. The run-in period of four weeks will allow the systems to be properly set up and tested before the trial commences. Time has been built into the study to allow practice staff to try out the systems hands-on before patient recruitment and data collection begin.

#### Safety of patients and study personnel during the research interviews

It is acknowledged that involvement in research interviews where patients are asked to reflect on their health issues can, in some cases, cause distress. Should any such difficulties occur during a research interview or focus group, the researcher will offer support to the person involved. Should a patient appear significantly upset and at risk as a result of the interview, the researcher will (with permission) advise the patient’s GP of this distress and also encourage the patient to seek further support from the support network available to them. Should there be any concern that a patient is likely to cause harm either to themselves or another person, the patient’s GP would be notified immediately.

Since researchers will be conducting interviews alone with patients, potentially in patients’ own homes, it will be important to have safeguards in place to protect both patient and researcher. Criminal Records Bureau (CRB) checks will be performed on any researcher taking part to ensure that they are an appropriate person to be working with vulnerable adults. To ensure both researcher and patient safety, the Lone Worker Policy and buddy system designed by the Primary Care Research Group will be adopted by the study researchers. This provides a mechanism for ensuring that the exact whereabouts of researchers and patients at any time point during the research is known by a supervisor or buddy.

### Research governance

The Research Management and Governance Unit, Devon Primary Care Trust will act as the sponsor for the trial. The trial will be hosted in the Primary Care Research Group (Peninsula College of Medicine and Dentistry, Exeter), a research setting specialising in primary care and community-based research currently participating in MRC- and NIHR HTA-funded trials.

The Trial Steering Committee (TSC) and Data Monitoring and Ethics Committee (DMEC) have been constituted following relevant guidelines such as the MRC *Guidelines on Good Clinical Practice in Clinical Trials* and the HTA TSC/DMEC guidance notes. A Trial Management Group (TMG, chaired by the PI) has been formed to guide the strategic direction of the study.

This trial will not be liable for registration under the Medicines for Human Use (Clinical Trials) Regulations 2004. We will, however, ensure that this trial is registered with http://www.controlledtrials.com and assigned an ISRCTN number. Relevant trial documentation will be retained for 15 years.

## Trial status

Start date: 1 April 2010 (main trial)

Expected end date: 31 July 2013

Expected publication date: 1 October 2013

Status at time of submission of this article: recruitment ongoing

Funder: UK National Institute of Health Research Health Technology Assessment programme

## Abbreviations

A&E: Accident and Emergency; CCA: cost consequences analysis; CCDS: computerised clinical decision support; CONSORT: Consolidated Standards of Reporting Trials; CRB: Criminal Records Bureau; DMEC: Data Monitoring and Ethics Committee; ED: Emergency Department; GLM: generalised linear model; GP: General Practitioner; DNA: did not attend; EMIS: Egton Medical Information Systems; EQ-5D: EuroQol Group 5-Dimension Self-Report Questionnaire; HTA: Health Technology Assessment; ICC: intracluster correlation coefficient; ISRCTN: International Standard Randomised Controlled Trial Number; ITT: intention to treat; MRC: Medical Research Council; MREC: Multi-centre Research Ethics Committee; NGMS: new General Medical Services; NHS: National Health Service; UK: United Kingdom; NICE: National Institute for Health and Clinical Excellence; NIHR: National Institute for Health Research; PCRN: Primary Care Research Network; PCT: Primary Care Trust; PI: principal investigator; PSSRU: NHS and Personal Social Services Research Unit; RCT: randomised controlled trial; SAFER1: Support and Assessment for Fall Emergency Referrers trial; SF36: Short Form (36) Health Survey; TSC: Trial Steering Committee; WIC: Walk In Centre; TMG: Trial Management Group.

## Competing interests

The authors declare that they have no competing interests.

## Authors’ contributions

JC conceived of the study and participated in the design and co-ordination of its delivery and helped to draft the manuscript. NB participated in the design of the study, led the process evaluation element and helped to draft the manuscript (NB is partially supported by the National Institute for Health Research Collaborations for Leadership in Applied Health Research and Care. The views and opinions expressed in this paper are those of the authors and not necessarily those of the NHS, the NIHR or the Department of Health). CG participated in the design of the study, led the health economics element and helped to draft the manuscript. TH participated in the design of the study and co-ordination within the Warwick recruitment site and helped to draft the manuscript. VL participated in the design of the study and co-ordination within the Norwich recruitment site and helped to draft the manuscript. SR participated in the design of the study and helped to draft the manuscript. DR participated in the design of the study and helped to draft the manuscript. CS participated in the design of the study and co-ordination within the Bristol recruitment site and helped to draft the manuscript. RT participated in the design of the study, led the statistical analysis plan and helped draft the manuscript. EF co-ordinated the delivery of the study, participated in acquisition of data and helped to draft the manuscript. All authors read and approved the final manuscript.
